# An Assessment of the Performance of the PLUS+ Tool in Supporting the Evaluation of Water Framework Directive Compliance in Scottish Standing Waters

**DOI:** 10.3390/ijerph17020391

**Published:** 2020-01-07

**Authors:** David Donnelly, Rachel C. Helliwell, Linda May, Brian McCreadie

**Affiliations:** 1 The James Hutton Institute, Craigiebuckler, Aberdeen AB15 8QH, UK; Rachel.helliwell@hutton.ac.uk; 2 UK Centre for Ecology & Hydrology, Bush Estate, Penicuik, Midlothian EH26 0QB, UK; lmay@ceh.ac.uk; 3 Scottish Environment Protection Agency (SEPA), Inverdee House, Baxter Street, Aberdeen AB11 9QA, UK; brian.mccreadie@sepa.org.uk

**Keywords:** total phosphorus, lakes, reservoirs

## Abstract

Phosphorus is one of the main causes of waterbodies in Scotland being at less than good ecological status (GES) in terms of the water framework directive (WFD). In Scotland, there are more than 8000 standing waters, defined as lakes and reservoirs that have a surface area of more than 1 hectare. Only about 330 of these are monitored routinely to assess compliance with the WFD. The export coefficient tool PLUS+ (phosphorus land use and slope) has been developed to estimate total phosphorus (TP) concentrations in the unmonitored sites; modelled values are then compared to WFD target concentrations for high, good, moderate, poor, and bad status to assess compliance. These type-specific or site-specific targets are set by the regulatory authority and form part of a suite of physical, chemical, and ecological targets that are used to assess GES, all of which must be met. During development, the PLUS+ tool was applied to 323 monitored catchments and 7471 unmonitored catchments. The efficacy of the tool was assessed against TP concentrations observed in 2014 and found to perform well in the rural catchments. 51% of standing waters had the same modelled and observed WFD class (i.e., High, Good, Moderate, Poor, Bad), and a further 40% of standing waters had a modelled WFD class that was within one class of observed water quality. The tool performed less well in catchments with larger inputs of TP from urban sources (e.g., sewage). The greatest deviations between measured and modelled classes were explained by the shortage of information on wastewater treatment works, fish farms, migratory birds, levels of uncertainty in TP measurements, and the amount of in-lake re-cycling of P. The limitations of the tool are assessed using data from six well documented case study sites and recommendations for improving the model performance are proposed.

## 1. Introduction

Excessive inputs of phosphorus (P) are the main cause of eutrophication problems in lakes and reservoirs. These adversely affect the ecological status of water bodies by causing hypoxia (oxygen depletion), loss of biodiversity, and harmful algal blooms [1,2]. In many parts of the world, P inputs to water have risen throughout the twentieth century [3,4,5,6,7]. This is due to increasing discharges from point sources such as wastewater treatment plants (WWTPs) [8], aquaculture [9], and losses from diffuse sources such as agricultural land [3,10], septic tanks [8,11], and migratory birds [12]. In Europe, legislation to support the implementation of the Water Framework (WFD), Urban Waste Water Treatment (UWWT), and Habitats Directives has set waste water treatment and water quality standards for the P concentrations in discharges and standing waters to address this problem. In terms of the WFD, standing waters are defined as lakes and reservoirs that have a surface area of more than 0.5 km^2^ [13].

The ecological status of aquatic habitats is the key driver behind the WFD legislation and is scored on a scale from High (pristine) to Bad, with the intermediate classifications of Good, Moderate, and Poor [14]. The legislation is supported by a river basin scale approach that aims to achieve at least good ecological status (GES) and recognises the need for changes in land management, in addition to implementing simple effluent reduction measures [15], to meet water quality objectives. The original aim of the WFD was for all waterbodies to achieve ‘Good’ ecological status by 2015, although that deadline has now been updated to 2027 [16] reflecting the large size of the task.

Traditional approaches for assessing the contribution of total phosphorus (TP) to water from various sources have been based on the development of detailed, physically based models that predict changes in water quality in real time. However, these models are expensive to construct, difficult to calibrate for large scale assessment, and are challenging for environmental managers to adopt in practice. In contrast, more simple export coefficient-based models can be used in data poor catchments to highlight knowledge gaps and help design better management strategies. They can also provide evidence to support decision making. The export coefficient modelling approach aims to predict the nutrient loading to water as a function of nutrient export from each source within a catchment [17]. Whilst export coefficient models have been applied within a framework of geoclimatic regions in the UK [17,18,19] and elsewhere [9,20], this study is the first national scale application of an export coefficient tool (PLUS+) that uses current land cover and population estimates to derive TP loads and concentrations in relation to standing waters in Scotland. Here, we present for the first time:

(a) Evaluation of the current TP status of standing waters in Scotland
(b) Description of the PLUS+ tool and its preliminary application to 323 monitored catchments, and 7471 unmonitored catchments.
(c) Evaluation of PLUS+ performance compared to observed data
(d) Assessment of the importance of TP in terms of non-compliance with the WFD
(e) Sensitivity analysis
(f) Evaluation of the effectiveness of the tool when applied to selected catchments that receive TP predominantly from sources other than the land runoff.

The PLUS+ tool was developed in collaboration with environmental managers using data on WFD status of monitored standing waters. The performance of PLUS+ was deemed sufficiently good in the development phase to apply it to 7471 unmonitored catchments. This larger number of sites allowed a preliminary assessment of the catchment typologies that are most at risk of failing to meet good ecological status (GES), thus providing a greater understanding of source apportionment of TP, and what measures and management actions should be put in place to reduce TP concentrations in all catchments.

## 2. Materials and Methods

### 2.1. Study Area

**Figure 1:**
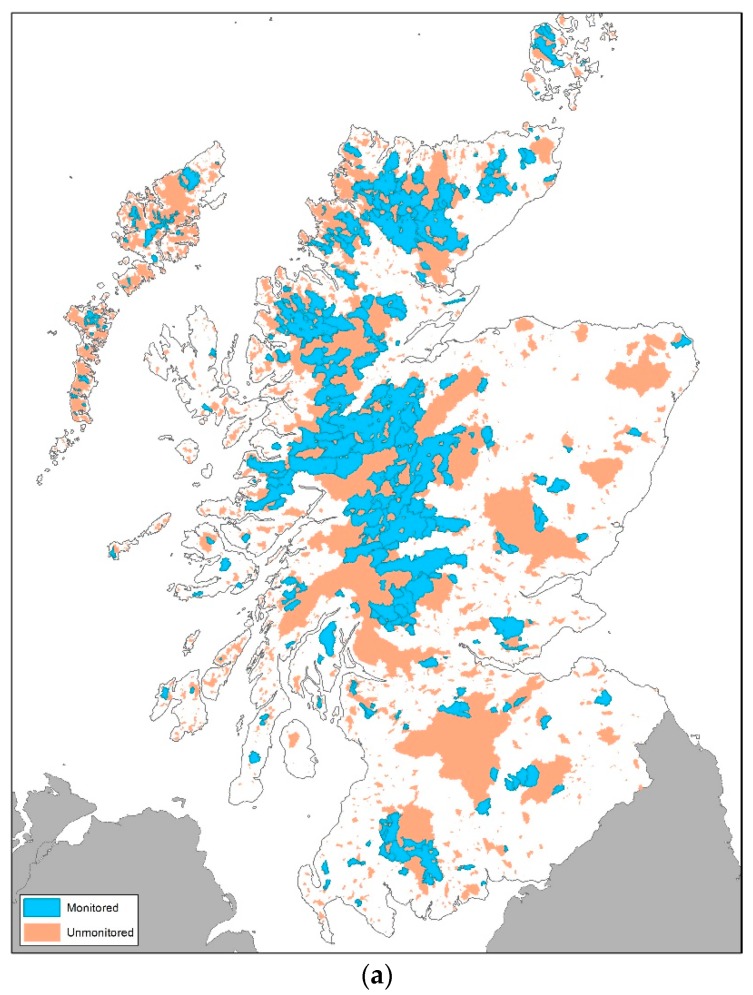

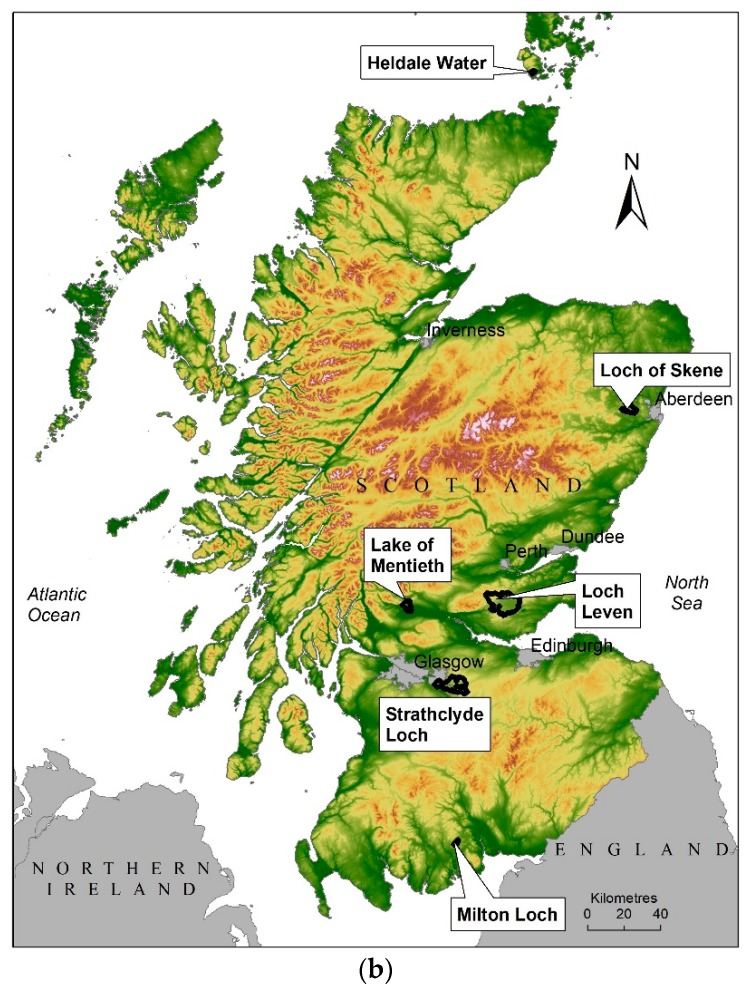
(**a**) Catchments of the 323 monitored lakes and 7471 un-monitored sites. (**b**) Topographic map of Scotland showing the locations of six case study sites.

The Scottish Environment Protection Agency (SEPA) monitor more than 330 standing waters (i.e., lakes and reservoirs >0.5 km^2^ in surface area) as part of their commitment to the EU WFD and national monitoring strategies. Following a check for completeness, 323 standing waters were included in this assessment. Figure 1a shows the location of monitored and unmonitored sites across Scotland and the location of the 6 case study sites. The diversity of the topographic area across which they are distributed is shown in Figure 1b. Scotland may be described as being divided topographically into upland and lowland regions, which broadly follow an east–west divide. The upland climate is cool and wet, with an average annual rainfall of approximately 2000 mm. Soils throughout Scotland are highly variable, and the distribution of various soil types is influenced to a large extent by climate, topography, and parent material. Many upland soils are nutrient poor, stony and coarse textured, or with high organic contents. Such soil and geological conditions can impose severe restrictions on land use. The main land cover in the uplands is heather moorland and semi-natural acid-grassland, which is largely sustained by low-intensity sheep grazing, and forestry (commercial and semi-natural). The eastern coastal belt and the central lowlands are the principal agricultural areas. The east coast is much drier than the uplands, with some parts receiving only 550 mm of rain per year. The soils in southern Scotland are complex and dominated by podzols and brown earths. These relatively flat lowland regions are highly productive and have a richer nutrient status than the uplands and benefit from organic and inorganic fertiliser additions. Significant urban areas include Glasgow, Edinburgh, Aberdeen, Dundee, Inverness, and Perth with scattered rural towns and settlements in the rural areas to the east and Scottish Borders.

In addition to the 323 standing waters in the model for which measured data were available, PLUS+ was also applied to 7471 unmonitored standing waters and six lowland case studies (Lake of Menteith, Central Scotland; Strathclyde Loch, West Scotland; Loch Skene, North East Scotland; Loch Leven, Fife- East; Heldale Water, Orkney; Milton Loch, Dumfries) that receive TP predominately from sources other than the land (Section 3.4).

### 2.2. Description of PLUS+

PLUS+ is a development of the Phosphorus Land Use and Slope (PLUS) tool. PLUS was developed by the Macaulay Land Use Research Institute in the 1990s in association with the Forth River Purification Board and with funding assistance from Scottish Natural Heritage. In conjunction with a hindcasted national land cover (ca. 1850) dataset, PLUS attempted to discern the relationship between anthropogenic drivers of nutrient enrichment, especially TP and the trophic status of standing waters in Scotland. This was carried out for 170 standing waters and delivered robust results. There was interest in using the tool to undertake further assessment of standing water TP concentrations and how these are affected by changes in diffuse and point sources of TP. However, the PLUS tool had several limitations. It was based on out of date geographic information system (GIS) technology, it lacked a user-friendly interface, and it had no facilities for maintaining records of simulations in a central database. PLUS+ has been developed to address these issues while continuing to use the export coefficients that were calibrated within the original version of PLUS [21].

PLUS+ is an interactive software tool written in visual basic for applications (VBA) for Esri ArcMap using ArcObjects, the use of which is described in an accompanying handbook [22]. The VBA forms and code, and the required data are available in a GitHub repository [23]. As PLUS+ has been coded to take advantage of the GIS environment provided in ArcMap this does mean that it can only be used within the ArcMap GIS application. The primary purpose of PLUS+ is to calculate TP concentrations and loads exported from land and other sources to standing waters in Scotland. In addition, PLUS+ has a scenario mode allowing users to model the impact of proposed or theoretical changes in land use, climate, and aquaculture on TP and WFD status. A more detailed description of the PLUS+ tool and the calculations used is given in the electronic Supplementary Information Sections S1–S3.

PLUS+ uses WFD water quality breakpoints and the red-amber-green traffic light system (Supplementary Information Section S5) that is currently used by SEPA to highlight how close a water body’s TP concentration is to a water quality boundary (e.g., between moderate and poor) and the capacity of that water body to accommodate additional P inputs (e.g., as part of a fish farm proposal) before the status is downgraded to a lower category. In PLUS+ the WFD status is calculated by comparing the modelled concentration to a database of water quality breakpoints Supplementary Table S3 and Supplementary Figure S3. Breakpoints were determined by SEPA from reference TP levels that were calculated using the Morpho-Edaphic Index (MEI), the ratio of total dissolved solids in lake water to the mean depth of the lake and the boundary Ecological Quality Ratios [16].

**Figure 2:**
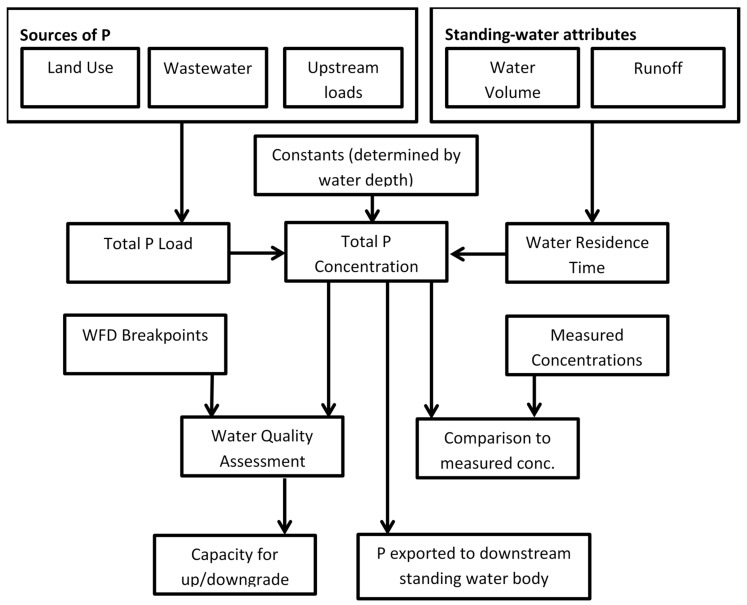
Schematic diagram of the main stores, processes and pathways included in the PLUS+ tool.

PLUS+ uses data tables in ArcMap that define and quantify the sources of TP and describe the characteristics (depth, area) of 8030 standing water bodies in Scotland (Supplementary Information Section S6). The inputs and outputs are summarised in Figure 2.

### 2.3. Sources of P included in PLUS+

#### 2.3.1. Land Cover

The tool incorporates TP exports from land cover and non-land-based sources. Diffuse loadings of TP from land are an important component of the PLUS+ tool. An analysis of existing available land cover maps (i.e., Land Cover of Scotland 1988 [24], (LCS88) with a minimum mapping area of 0.25 hectares and the Land Cover Map 2007 [25] with a minimum mapping area of 0.5 hectares) highlighted a number of issues, mainly associated with evolving agricultural practices and woodland and urban expansion. To overcome these problems, a new land cover map was produced in 2015 based on contemporary spatial data from Ordnance Survey MasterMap, the National Forest Inventory (Forestry Commission), the Integrated Agricultural Census Scotland (IACS, Scottish Government), GreenSpace Scotland, and LCS88 (for semi-natural, non-forested environments). Land cover from these inputs was summarised into 19 categories, each with an associated range of TP loss coefficients (Table 1). In addition, each land cover area was categorised by a slope classification of low, medium and high, based on thresholds shown in Table 1. The slope classification was determined using Ordnance Survey elevation data.

Phosphorus export coefficients were assigned to each slope category of each land cover type. The default export coefficients used in PLUS+ are those calculated by Ferrier et al. [21] by back calculating the total TP load into each waterbody from the best available estimate of TP concentration in the waters. These form the basis of the PLUS+ tool’s ability to determine the spatial loading/concentration of TP within a catchment, within a range of predicted maxima and minima.

Export coefficients were highest for land cover types that included (a) significant amounts of TP from industry/urban; (b) high inputs of organic/inorganic fertiliser/manure (arable/improved grassland) and/or (c) where there was land disturbance caused by forestry operations. There was a greater export of TP from steeper slopes than gentler slopes (Table 1).

#### 2.3.2. Waste Water Treatment Works and Septic Tanks

Effluent from waste water treatment works (WWTW) is the principal point source of TP to surface waters, although to a lesser extent, diffuse sources from septic tanks are also important. In the absence of complete data from WWTWs and septic tanks, a method was developed to estimate per capita TP export from urban residential properties equivalent to that which could be expected from WWTWs to surface waters. Population census data from 2011 was combined with data zone boundaries (Scotland’s standard small-area statistical unit, [26]) and the locations of residential properties to create an updated spatially explicit population model for Scotland (Supplementary Information Section S4). This was used to create a model of urban (WWTW) and rural (septic tank) sources of TP. All waste water from urban populations was assumed to reach standing waters via WWTWs (the locations of WWTWs were not identified in the tool) and a per capita TP load rate (loss coefficient) was assigned a default value of 0.9125 kg P per capita [21]. The effect of P stripping technology in lowering P discharges was not considered. Waste water from rural populations was assumed to enter a surface water body via septic tanks and a per capita P load rate (loss coefficient) was assigned as a default value of 0.25 kg P per capita (ibid.). Only those tanks within the riparian zone of surface waters were included in the study. Export of P from the population within a catchment is routed via the upstream network to the standing water body using a cascading principle, which transports flow between sub-catchments ([22], (Supplementary Information Section S6, Supplementary Equation (S4)).

#### 2.3.3. Other Sources of Phosphorus

Sources of P that were not included in this study include fish farms and birds, especially geese, and atmospheric deposition. Unlike the systematic records held for marine fish farms (via Scotland’s Aquaculture website [27]), there are no publicly accessible official records of the location, size, nutrient input, or level of production of freshwater fish farms in Scotland. In addition, as roosting geese are known to contribute to the TP budget of standing waters along their migratory path, this input was considered as part of the Loch Skene case study. However, data availability was limited at a national scale.

### 2.4. Input Data to PLUS+

PLUS+ makes use of the following data sets:

(a) Mean annual TP concentrations for 2014 from SEPA’s routine monitoring campaign of standing waters. This data has 334 records, however, eight records have a TP concentration of zero (and zero confidence), and three more have a waterbody ID that is not in the PLUS+ database, leaving 323 waterbodies with measured TP concentrations.
(b) Meteorological Office 5-km climate grids from 2010 were used to generate discharge (to calculate TP loads) and using a water balance model [28,29]. Meanwhile, potential evapotranspiration was estimated using the FAO56 modified Penman–Monteith methodology [30]. The water balance model also includes soils information from Hydrology of Soil Types (HOST) classes [31].
(c) Land cover and associated TP exports as described in Section 2.3.1.
(d) Specific data on bird numbers (case study sites only).
(e) TP breakpoint data with TP concentration thresholds corresponding to the WFD status, supplied by SEPA.
(f) Sub-catchment boundaries corresponding to the standing waters in the model. There are 8030 standing waters for which PLUS+ will calculate the TP. However, it is not possible to assess 236 of these standing waters against WFD status as they are not included in the TP breakpoint data. This means that there is a total of 7471 unmonitored waterbodies in PLUS+ which may be assessed against WFD status.

### 2.5. Phosphorus Load and Concentrations

The P loads and concentrations are calculated according to the method described by OECD, [32] and in the Supplementary Information S2.

### 2.6. Sensitivity Testing

The development and refinement of the PLUS+ tool included the testing of model sensitivity to varying inputs. Sensitivity testing was conducted with the fixed range of export coefficients [21]; with changes in discharge values; and with two different land cover models to assess the influence of land cover types and their P exports on TP. During sensitivity testing each input parameter was adjusted in turn whilst holding all other parameters constant. The net effect on model output was assessed for each parameter to determine which had the greatest effect on model output.

#### 2.6.1. Export Coefficients (EC)

The land cover export coefficients for PLUS+ were developed with minimum, median and maximum values to take some account of the expected uncertainty inherent in using expert judgement to estimate the values, and to reflect anticipated variations in the landscape. These are listed in the Supplementary Information S7, Table S4. For each land cover type, a minimum, median, and maximum export coefficient was determined for each of the three slope categories used in the tool. The ratio of maximum to minimum of P export coefficient values range from 1.875 (grasslands, steep slope, maximum export coefficient = 0.15, minimum = 0.08) to 7.5 (wetlands, flat, maximum export coefficient = 0.15, minimum = 0.02). The default setting of the PLUS+ tool is the median value for each land cover/slope class. However, for this study, the tool was run using all three sets of export coefficients (i.e., median, minimum and maximum values) to investigate its sensitivity to the land cover export coefficients. The P concentrations resulting from this varying of inputs are summarised in Table 2.

In relative terms, a change in land cover export coefficient has more effect in catchments with low overall P concentration. These catchments are likely to have a small population-based TP. Those catchments with the highest TP concentration are those with the largest population so that the contribution of land cover sourced P makes a lower contribution to the TP.

#### 2.6.2. Discharge

The sensitivity of PLUS+ to changes in discharge was tested by arbitrarily varying the discharge between plus and minus 10% of the standard modelled discharge in steps of 5%. The resultant TP loads were compared to the baseline values (Table 3). This analysis demonstrated a small and statistically insignificant decrease in TP concentration with increased discharge across the 8030 standing waters.

#### 2.6.3. Land Cover

To test the sensitivity to changes in P concentration and land cover, a simple approach was adopted whereby P valves derived from a contemporary land cover dataset (2015) were compared to those of the Land Cover of Scotland 1988 [21] across 8030 standing waters. The EC of the key land cover types remained the same, but the proportional coverage had changed in response to drivers such as nitrogen deposition, urban expansion, land management and agricultural policy over the 27 year period. A more detailed description and a table of the comparison of the export coefficients associated with a change in land cover is included in Supplementary Information S8.

The largest observed change in land use between 1988 and 2015 was from heather moor to coarse grassland, covering an area of 1.4 M ha. It is well understood that nutrient enrichment of upland heaths from atmospheric deposition of nitrogen has increased the dominance of grasslands in the UK [33]; however, this figure is high and may represent some uncertainty in the IACS classification used to construct the land cover map for 2015 compared to that for 1988. Heather moorland and coarse grassland have the same P export coefficient and so this change does not affect calculated TP concentrations. The area of the second largest land cover class (improved grassland, ~0.9 M ha) remained unchanged. Note that this class reflects the dominance of diffuse livestock derived TP loadings (EC 0.47) [34]. Results from the sensitivity analysis showed that the net concentration of TP exported to standing waters increased slightly across the 8030 catchments in 2015 (Table 3), reflecting a number of complex land cover changes, including the larger area (0.6 M ha) of coarse grassland in 2015 (EC 0.09) compared to the previously mapped area of blanket bog/peatland (EC 0.03). It has been proposed that the increase in TP from this particular land use change was the result of widespread drainage, grazing and/or fertilisation of blanket bog and peat in recent decades [35,36,37].

Results presented in Section 3 reflect the use of P export coefficients based on the catchment specific slope and contemporary (2015) land cover categories.

## 3. Results

### 3.1. Observed TP Concentrations in Scottish Standing Waters

It is generally accepted that the trophic status of a standing water can be categorised according to its P concentration. Standing waters with P concentrations below 10 µg P/l are classified as oligotrophic, while those between 10 and 20 µg P/l are mesotrophic, and those with concentrations exceeding 20 µg P/l are classified as eutrophic [38]. Most standing waters in Scotland are nutrient poor and therefore oligotrophic. Of the standing waters currently monitored by SEPA, 75 out of 323 in this study have concentrations of <5 µg P/l, and 183 had TP concentrations of <8 µg P/l. Approximately 20% of standing waters had significantly higher TP concentrations; these are classed as mesotrophic or eutrophic. Standing waters can be naturally mesotrophic or eutrophic, however standing waters within these classes that exhibit higher concentrations compared to the baseline fail to meet water quality standards and GES. These catchments are located predominantly to the east and central lowlands of Scotland, where land is intensively managed for agriculture and significant amounts of TP are exported to standing waters from WWTWs.

### 3.2. Validation of PLUS+ TP Concentrations against Measured Data

**Figure 3:**
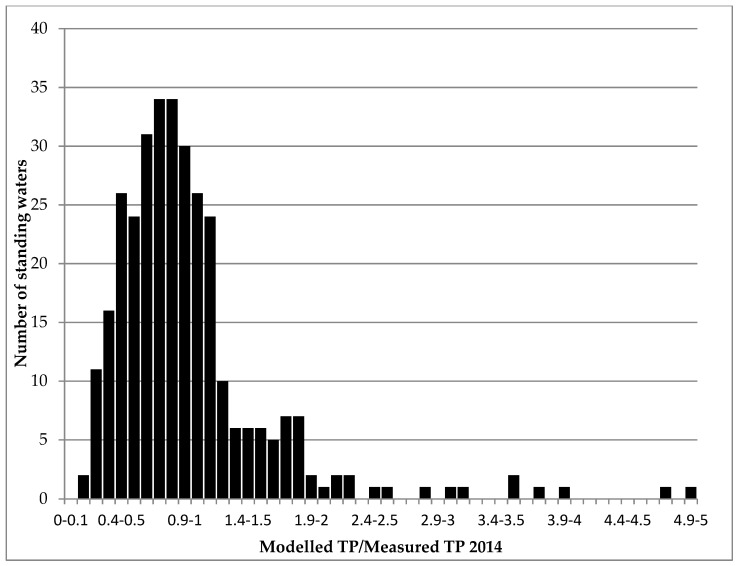
Frequency distribution of the ratio between TP concentrations derived from PLUS+ and those measured in 323 Scottish catchments (2014).

Model performance was assessed against 323 observed values for TP concentrations. Given the national land cover, slope, and population datasets used in this application, and the broad land cover categories used for the export coefficients, PLUS+ performed reasonably well (Figure 3). The median value of modelled TP divided by measured TP was 0.85 (n = 323). Modelled TP concentrations matched measured TP at 30 sites, and modelled TP was within 20% of the measured values at 148 sites. There was some error associated with the current parameterisation of PLUS+, as it did not appear to accurately model TP equivalent to that exported from WWTW, particularly in urban catchments with large estimated populations. However, when accurate WWTW information was included in a test catchment the accuracy of the modelled result improved significantly (see Strathclyde Loch case study below). There was also some uncertainty in the modelled TP for catchments known to have farmed livestock. Although the export coefficient for improved grassland is set to account for TP from livestock, quantification of national livestock numbers was not part of this study, making modelled estimates difficult to verify. In general, these observations (waste water/livestock) are considered to be responsible for the discrepancy between modelled and measured TP, as reflected in the tails of the distribution in Figure 3.

There was a clear typological element to the model performance. PLUS+ performed well in catchments located in rural areas where the dominant land cover was arable and the population density was low. Similarly, modelled TP versus measured TP showed a strong correspondence in catchments in the Scottish Highlands and the Border region, where land use is dominated by semi-natural habitats (heathland, peatland and coarse grassland and forestry). In these situations PLUS+ is capable of calculating accurate annual export fluxes of TP.

PLUS+ overestimated TP in standing waters located in catchments with large populations in the central belt and near significant towns along the east and north east coast of Scotland. In general, standing waters with a high modelled to measured TP ratio (Figure 3) represent catchments with a large population and, thus, a poor representation of the flux of P from waste effluent within the PLUS+ parameters. In Section 3.4, we investigate a number case studies where PLUS+ failed to achieve good results and propose methods to improve model performance.

### 3.3. National Assessment of Compliance with the Water Framework Directive

#### 3.3.1. Total Phosphorus Status of all Standing Waters in Scotland

**Figure 4:**
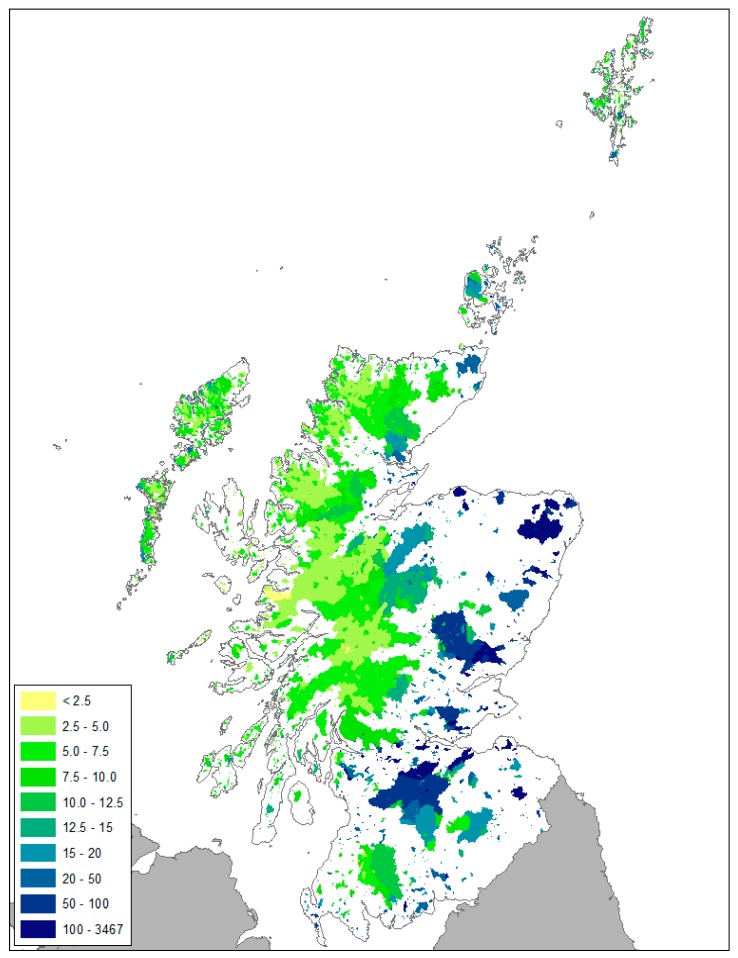
Catchments of all 8030 standing waters showing their estimated in loch P concentrations (µg P/l).

**Figure 5:**
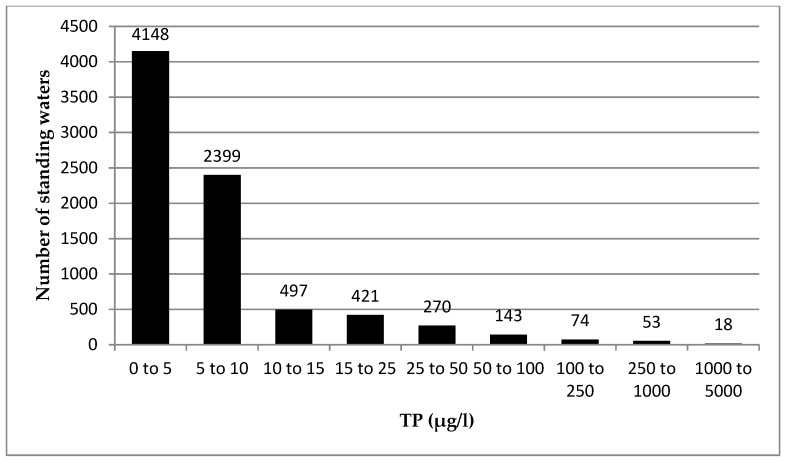
Frequency distribution of modelled TP concentrations in 8030 standing waters throughout Scotland (based on data from 2014). Note that seven standing waters were calculated to have zero TP concentration and are not shown above.

Noting the strengths and weaknesses of this approach, the PLUS+ tool was used to assess, for the first time, the current status of all standing waters in Scotland as legislated under the WFD. The spatial and numerical distribution of modelled standing water TP concentrations for all 8030 sites is presented in Figure 4 and Figure 5 respectively. The highly skewed distribution demonstrates that, at a national scale, 51% of standing waters in semi-natural landscapes are oligotrophic and have a TP concentration <5 µg P/l. Of concern are the standing waters with a TP concentration in the class 5–10 µg P/l (30% of all standing waters in Scotland). Given the greater confidence in the model performance for these catchments with a rural/agricultural typology, the results indicate with some confidence that there are 2399 standing waters with a TP concentration close to the good/moderate threshold for ecological status (broaching mesotrophic status). Mesotrophic lakes are sensitive to artificially increased levels of P [39], so any increase in TP export to these systems from changes in land use, climate, and waste water could have serious implications for the future compliance of waterbodies. There is a large upper range of modelled concentrations in PLUS+ (Figure 4 and Figure 5), including for example values over 3600 µg P/l. However, in the sub-catchments with concentrations exceeding 1000 µg P/l, all cases have at least 95% of their P load calculated to be associated with P from their urban population, most of which will be treated in WWTW. For standing waters with concentrations exceeding 250 µg P/l, at least 90% of the P emanates from the urban population, and, in the remaining cases, upstream sources of P from the urban population in adjacent catchments provide the majority of P. Clearly, in the absence of comprehensive and accessible information on TP exports from wastewater sources, further investigation into a national scale method for estimating TP export from these sources is necessary.

#### 3.3.2. Compliance/Non-Compliance with the WFD Based on Modelled and Measured TP Concentrations

**Figure 6:**
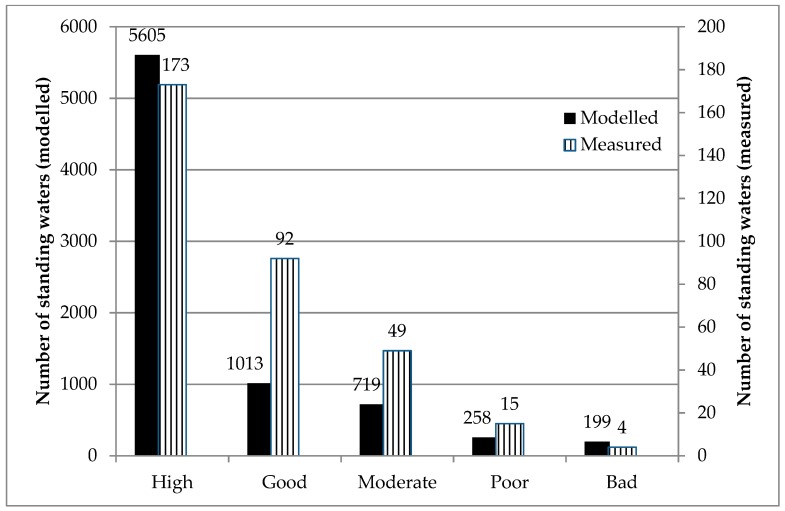
WFD status of 7794 modelled standing waters and 333 measured standing waters (Note that, due to data limitations, not all standing waters for which concentrations are calculated will have a WFD status, and that although the table shows all 333 SEPA reported classifications, only 323 of these can be modelled in PLUS+).

**Figure 7:**
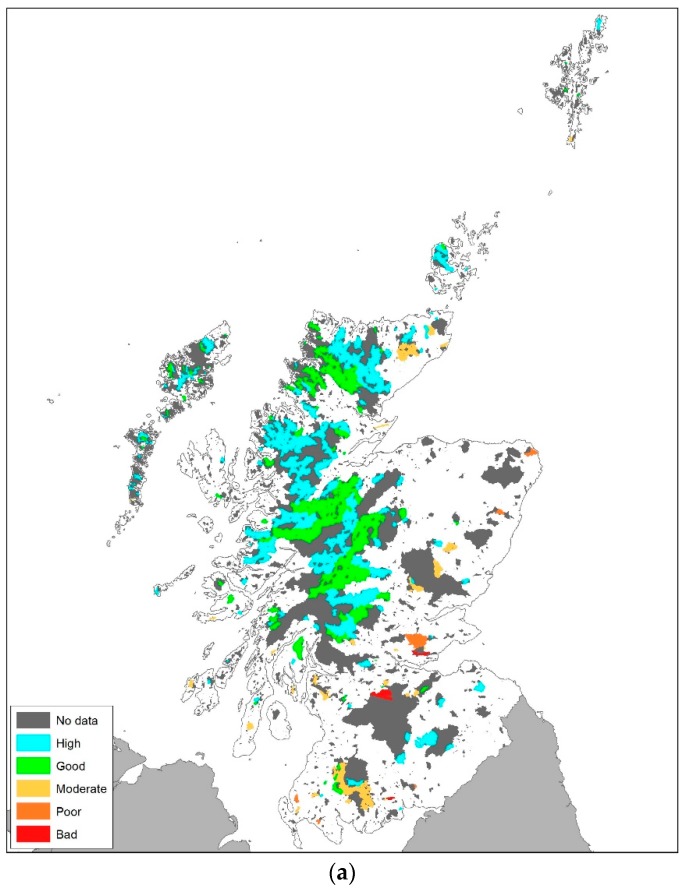

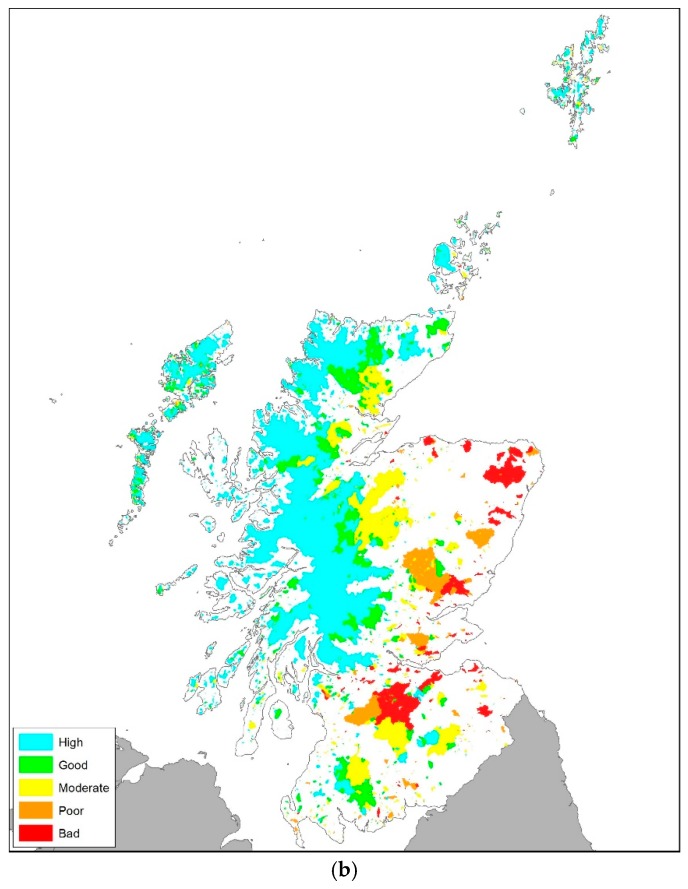

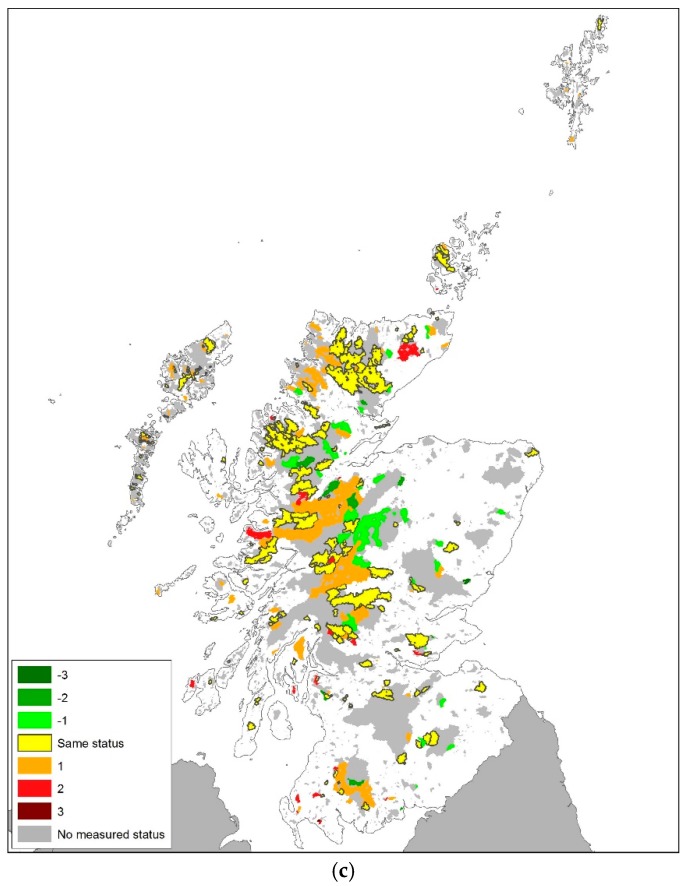
(**a**) WFD water body status based on 323 standing waters monitored by SEPA. Un-monitored catchments are shown in dark grey (No data). (**b**) WFD water body status based on modelled TP at 7794 standing waters (not all water bodies in the database have WFD thresholds that allow status assessment). (**c**) Difference between modelled status and measured status as described in Section 3.3.3 (e.g., 0 (yellow) = same modelled and measured water body class; −1 (light green) modelled = Good and measured = High (Table 4).

Based on modelled TP concentrations and catchment typologies, standing waters were assigned to a GES class (Figure 6 and Figure 7a,b). In total the majority (6618 out of 7794) of modelled standing waters for which a WFD status can be calculated meet the WFD target for good ecological status (GES above the good/moderate boundary). Similarly, of the 333 monitored standing waters, 265 have sufficiently low TP concentrations to be classified as at a high or good ecological status. The spatial distribution of standing waters with low TP concentrations and good ecological status is predominantly to the north, north-west and southern uplands where land use is mainly nutrient poor, semi-natural systems (Figure 7a). The TP status reflects the low export coefficients for coarse grassland, blanket bog and peat (Table 1). The majority of standing waters monitored by SEPA are located in these low nutrient areas (Figure 7a), as there are considerably fewer standing water bodies in the northeast and Border regions of Scotland. It is as important to monitor standing waters with low nutrient concentrations as it is to monitor more eutrophic systems, as they are good indicators of environmental change. As expected, a considerable number of standing waters currently fail to meet GES (1176 modelled and 68 measured), (Figure 6). The majority of standing waters that fail to achieve GES are situated in the more agriculturally intensively managed areas to the northeast, Fife and the Borders of Scotland, and/or are located in catchments with large urban populations (significant export of TP from waste water) (Figure 7a).

#### 3.3.3. Comparison of Modelled and Measured WFD Class at 323 Surface Waters

Modelled concentrations were compared with threshold values for GES on a catchment by catchment basis to determine the number of standing waters that met the objectives of the WFD. Of the 323 monitored catchments modelled in PLUS+, 175 (54.2%) had the same modelled and observed WFD class (i.e., high, good, moderate, poor or bad), (Table 4). In a further 108 (33.4%) of catchments, PLUS+ modelled WFD class was within +/− one water quality class (e.g., measured = High, modelled = Good). The WFD class of 35 (10.8%) catchments had measured and modelled assessments which were two intervals apart and five which are three intervals apart. A comparison of WFD water body status (modelled and measured) in a spatial context is shown in Figure 7c. The modal comparison result is measured = High and modelled = High with 146 (45.2%) catchments. The second most common comparison is measured = Good, and modelled = High with 53 (16.4%) catchments.

### 3.4. Case Studies

**Figure 8:**
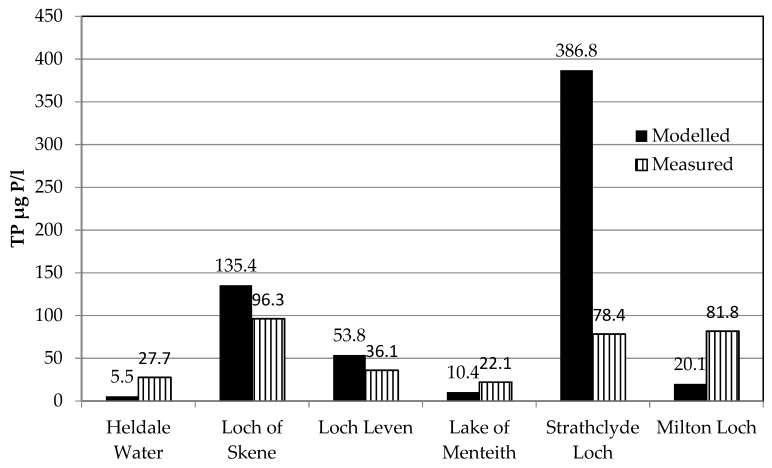
Comparison of measured and modelled P concentration in the selected case study catchments.

Six case studies were selected from across Scotland (Figure 1) to represent: (a) standing waters where PLUS+ failed to predict the correct WFD class, (b) a range of catchment and lake typologies, and (c) TP concentrations. This assessment assisted the evaluation of model performance where results were unsatisfactory. The comparison between the case study measured and modelled concentrations is shown below in Figure 8.

Heldale Water is located 1 km inland on the western edge of the island of Hoy (Orkney Islands), to the far north of Scotland (Figure 1b). The measured and modelled standing water TP concentration was 27.7 µg P/l (corresponding to a load of 211 kg/yr, WFD status Moderate or Poor) and 5.5 µg P/l (corresponding to a load of 29 kg/yr, WFD status High) respectively, as shown in Figure 8). Inspection of recent aerial photographs confirms that the land cover used in PLUS+ was correct, comprising coarse grassland, heather, blanket bog and peatlands. In agreement with the model inputs, there are no settlements or sewage inputs in this catchment. Further investigation of the low modelled TP concentration highlighted two potential sources of P that were missing from the simulation: a) TP from the large population of seabirds that frequent the catchment (Pers comms. avian ecologist L. Gilbert, University of Glasgow), and b) a relatively minor input, but noteworthy input of aerosols of P carried by the prevailing westerly winds from marine sources [40].

The total P concentration was over-estimated at Loch of Skene in Aberdeenshire (Figure 8). The measured concentration was 96.3 µg P/l (WFD status = Poor), and the modelled concentration was 135.4 µg P/l (WFD status = Bad). More detailed analysis of the waste water treatment process in this catchment revealed that all of the sewage produced is pumped to a WWTW outside the catchment, and that there are no contributions of TP from WWTW to Loch of Skene. In the model, a significant load of TP from waste water accounted for most of the imbalance between the modelled and measured TP. Additionally, it is known [41] that large numbers of migrating geese visit the catchment each year and that the annual P load calculated from this source is approximately 450 kg /yr. Revising the PLUS+ tool with this new information reduced the calculated P concentration in the water to 60.4 µg P/l, resulting in the same WFD status for measured and modelled TP.

Loch Leven in Perth and Kinross (Figure 1) is the only Scottish standing water to have been included in the original OECD study [32] that underpinned the TP modelling framework for PLUS+. Loch Leven is one of the “Shallow Lakes and Reservoirs” for which a denominator of 1.02 (“a” in Supplementary Equation (S1) and an exponent of 0.88 (“b” in Supplementary Equation (S1)) were derived. However, within PLUS+ a threshold of 3.0 m for water body depth was chosen to govern the choice of denominators and exponents from the OECD study. As Loch Leven has an average depth of 4.5 m, it was assigned the denominator and exponent from the combined results of the whole OECD study and not the values calculated specifically for “Shallow Lakes and Reservoirs”. Tests running the PLUS+ tool for all water bodies indicate that a threshold of 3.0 m for the assignment of model parameters appears to have been well chosen, when the calculated TP concentrations are compared to the measured concentrations. It should be noted that, in at least one other study [42], only the combined denominator and exponent were used, and that water depth was not included to determine these model parameters. Using the standard PLUS+ parameters, Loch Leven has a modelled TP concentration of 53.8 µg P/l (WFD status Poor). The measured value is 36.1 µg P/l (WFD Status Moderate). Modifying PLUS+ to use OECD denominator and exponent values for shallow lakes and reservoirs for Loch Leven (and its contributing standing waters which have similar depths) the modelled TP is reduced to 45.9 µg P/l, and therefore shares the same WFD status of Poor as derived from the observed data.

The Lake of Menteith is situated between the highlands and central lowlands (Figure 1b) and is the waterbody used to calibrate the original PLUS tool in the 1995 research [43,44]. The PLUS+ modelled concentration of 10.4 µg P/l (WFD status High) corresponds to a P load of 399 kg/yr. The measured concentration in 2014 of 22.1 µg P/l (WFD status Moderate) corresponds to a load of 1006 kg/yr. Table 5 shows the sources and annual loads of TP that were used in the original PLUS application [44]. The contribution of P from land cover in PLUS+ is 329 kg/yr and substantially lower than the 503 kg in the 1995 study. It is notable that an area of arable land (with a high P export coefficient) is included in the 1995 study. Inspection of 2015 Scottish Government farm data indicates that this has now been replaced by land cover types associated with a lower P export coefficient (predominantly coarse grassland). Adding the fish, birds and deposition loads described in the 1995 study to PLUS+ produces a revised concentration of 15.0 µg/l (WFD status Good) and a load of 626 kg P/yr. Marsden et al. [43] reports a long term mean P concentration in Lake of Menteith of 21 µg P/l (WFD status Moderate). Given the known characteristics of the lake, this would require a P load of 951 kg/yr, which is towards the upper end of the range described in the 1995 study, and indicates that median loads may underestimate the true P loads to the system.

The measured TP concentration in Strathclyde Loch, central Scotland was 78.4 µg P/l (WFD status Poor), while the modelled concentration was 386.8 µg P/l (WFD status Bad). This is one of the largest discrepancies between modelled and measured data in PLUS+. When the standard PLUS+ method for simulating TP from WWTWs is applied (i.e., a load of 0.9125 kg P per capita for the urban population within the sub-catchment, [S1]), the load from this source is 58,028 kg of P (91.5% of the subcatchment total). For this research, Scottish Water (responsible for the WWTW) supplied information on the effluent discharge in this sub-catchment. This information showed that effluent was being discharged to rivers, not to the standing water as assumed in the standard PLUS+ application. Including these WWTW data in the model, and adjusting the per capita loads appropriately, reduces the calculated TP concentration in the standing water to 83.7 µg P/l, which is within 7% of the latest measured concentration.

PLUS+ significantly underestimates the TP concentration in Milton Loch, Dumfries (Figure 1b). The modelled TP is 20.1 µg P/l (WFD status High), which is less than one-quarter of the measured concentration of 81.8 µg P/l (WFD status Poor). This equates to a discrepancy in total P load of 558 kg/yr. This catchment has no upstream contributions of TP and visual inspection of recent aerial photography indicates that the land cover is not different to the land cover model. A theoretical scenario with the PLUS+ model set so that all land cover belongs to the class with highest P export coefficient (arable) increases the P concentration to 37.4 µg P/l, still less than half of the measured concentration. There is no urban population and only a small rural population lives in this sub-catchment. So more than 500 kg of annual TP loading remains unaccounted for. Algal blooms are mentioned in an archaeological survey from 2003, indicating that nutrient enrichment is a problem at this site [45]. An improved understanding of the hydrology of the catchment and of the waterbody may help explain the high measured TP levels at this site. For example, low flushing rates and frequent releases of P from the sediments [46] may be responsible for an accumulation of P in the system.

## 4. Discussion

The Scottish Environment Protection Agency (SEPA) currently reports the status of standing waters to the European Commission as part of Scotland’s commitments to the WFD. Scotland’s current assessment of lake status is based on monitoring data collected from 330 standing waters. However, these represent only 4% of the total number of standing waterbodies in this country. So, a management tool is needed to provide likely status information for unmonitored waterbodies and to inform the implementation of a programme of measures to maintain and improve our water quality across all lochs.

The PLUS+ tool described in this study is compared, below, to several other models that are available to calculate the losses of TP from land to water across the UK. These include a three-tiered risk assessment tool [47], the SAGIS tool [48], the ‘ADAS’ tool [49], and the long term large scale (LTLS) model [50].

The three-tiered risk assessment tool [47] is a relatively simple model that takes an export coefficient approach to determining P inputs to lakes and their consequent water quality. The model increases in complexity from Tier 1 to Tier 3. At Tier 1, overall risk of a lake failing WFD P targets is assessed using simple export coefficients that are based on land cover, population density and animal numbers. At Tier 2, this P pressure is linked to the likelihood of that P reaching a standing water. At Tier 3, a Phosphorus Indicators Tool (PIT) that is based on a semi-distributed model is used to estimate the source, transport, and delivery of P to water from diffuse sources in more detail. The approach of PLUS+ is broadly comparable to Tier 1 of the 3-tier tool [47]. The main differences are that PLUS+ is an interactive tool and that it has the capacity to model interconnecting catchments simultaneously (e.g., the 184 sub-catchments in the Loch Ness system). PLUS+ also takes account of flows and residence times within the catchment, which the Tier 1 tool does not, and it runs at a much higher spatial resolution (50 × 50 m) than the Tier 1 model (5 × 5 km). Accordingly, outputs are likely to be more accurate at the catchment/site specific level.

The source apportionment-GIS (SAGIS) tool [48] apportions loads and concentrations of chemicals, including P, to WFD water bodies. Although based on the process-based PSYCHIC model [51], which originally included dissolved and particulate forms of P, the operational version of SAGIS used by SEPA predicts soluble P loads and concentrations, only. Because it does not provide TP values, it cannot be used to assess WFD compliance in these waterbodies. This is an important difference between PLUS+ and SAGIS. SAGIS has been developed to predict concentrations of soluble P in rivers, as required for the WFD, but similar assessments for lakes need to be based on TP concentrations. So, although SAGIS modelling is more process based and complex than that of PLUS+, the outputs from the current version of the SAGIS tool are much less suitable for assessing the risk of lakes failing their WFD P targets than those from PLUS+. Also, the spatial resolution of SAGIS is lower (1 km × 1 km) than that of PLUS+ (50 × 50 m), potentially making SAGIS less accurate at a catchment/site-specific scale.

Like SAGIS, the ‘ADAS’ model [49] is also based on the field scale version of the PSYCHIC model [51,52,53]. In contrast to the above, the ADAS version calculates the delivery of both soluble and TP to the receiving water bodies. Again, the ADAS model operates at a lower resolution than PLUS+. However, being process-based, the ADAS model also requires more complex and much larger amounts of data than PLUS+, making it more data intensive and difficult to update.

Another method of deriving P inputs to Scottish lakes and predicting the resultant in-lake water quality was developed by the LTLS project (http://www.ltls.org.uk) [50]. This model comprises UK national-scale integrated modelling of the fluxes and stores of P within the landscape at a resolution of 5 × 5 km. The model combines simple process-based models to provide national-scale coverage [50] and is driven by readily available, national scale, data such as climate, deposition, land cover, agricultural practices, topography. While the model provides national scale consistency of methods and data, the disadvantage of using such a large-scale model is that it is not calibrated specifically to local conditions [50]. So, it will be less accurate at the local scale than a fully calibrated site or catchment specific water quality model, such as PLUS+. Also, the LTLS model also has a much lower spatial resolution (5 × 5 km) than PLUS+ (50 m × 50 m).

The methods developed for the PLUS+ tool were guided by the availability of national datasets. As with any large-scale modelling assessment, the quality and accuracy of data decrease as the scale increases. It is clear the current structure of PLUS+ and the supporting data require some refinement before PLUS+ can be used as an effective management tool for national scale assessments of water quality. The greatest deviation between measured and modelled WFD classes occurred in catchments with a large population, and where the method used to estimate sewage loads to standing water was limited by data availability. However, as shown in the Strathclyde Loch case study, where detailed information was available for inclusion in PLUS+, a much more accurate assessment could be obtained. Also, whilst a method was devised to quantify the amount of TP leached from septic tanks, there remains considerable uncertainty regarding the level of TP export from these systems, especially in relation to those situated at some distance from riparian areas [54]. In the current, national scale application of PLUS+, no consideration was given to the TP load to standing waters from operational fish farms. This is potentially a very important source of TP on a national scale, which is rarely quantified effectively in catchment level assessments of water quality. For example, based on data from 2015, 171,722 tonnes of salmonids are produced at 87 freshwater sites across Scotland [55]. Finally, it is difficult to assess the migratory paths of birds such as geese and to quantify the TP inputs to lochs from their guano at a national scale [12]. Whilst this was attempted for the Loch of Skene case study using local data, there remains considerable uncertainty in the quantification of annual geese numbers and TP loads to the aquatic and terrestrial compartments of a catchment.

Outputs from the PLUS+ model indicate that TP concentrations in standing waters are broadly related to land use and effluent discharges. In particular, catchments with intensive agriculture, such as those in the northeast of Scotland and the Borders, have higher predicted concentrations of TP than catchments in the Highland that have mineral-poor soils, low population densities and low intensity agriculture. Whilst the majority of standing waters in Scotland meet WFD GES targets, the PLUS+ tool suggests that there are a number of catchments where remediation measures need to be introduced. These measures include the stripping of P from wastewater at treatment works [8], improvements in land management practices in intensively managed areas [56], and the use of sensitive ground preparation techniques in forested areas. Our initial findings provide important evidence beyond SEPA’s monitored catchments of the number of catchments in Scotland that are potentially at risk of failing to meet GES, and this will have wider relevance to many stakeholder groups.

## 5. Conclusions

PLUS+ provides a good overview of the TP loads to lakes in Scotland and of the resultant P concentrations in those lakes. The performance of PLUS+ at SEPA’s monitored catchments was deemed sufficient for wider application to 7700 unmonitored catchments. This larger population of catchments allowed a preliminary assessment of the catchment typologies most at risk of failing to meet GES; providing a greater understanding of what measures and management actions should be put in place to reduce TP concentrations in monitored and unmonitored catchments.

The PLUS+ tool is more practical than some of the models outlined above, in that it needs less detailed data and less processing power than more process-based models. It is also of higher resolution than some of these models, making it potentially more accurate for site level determinations. Most importantly, the PLUS+ tool was developed by scientists and practitioners to ensure that the model was fit for purpose, and capable of meeting the operational requirements of the SEPA.

However, its performance could be improved at national scale if the following recommendations were followed:

- A national comprehensive and spatially explicit dataset of annual TP export from WWTWs should be included, with an indication of which standing waters they discharge effluent to (i.e., some WWTWs discharge effluent to standing waters in another catchment).
- The P losses from septic tanks to water need to be updated to reflect recent findings which concluded that discharges to water from these systems could be as high as 0.6 kg P/yr (per septic tank) [57].
- When spatial data of the locations of septic tanks is published, it should be included.
- The land cover map described in this study represents a collection of the best available spatial data for Scotland; however, it needs to be updated when more recent data become available, such as the more recently published Land Cover Map 2015 (LCM2015; https://www.ceh.ac.uk/services/land-cover-map-2015) and the next Countryside Survey for the UK.
- A national database of freshwater fish farm operations and their P exports to freshwaters needs to be developed and added to the PLUS+ tool
- The export coefficients within PLUS+, and their associated land cover classes need to be reviewed within an uncertainty framework.
- Given that TP concentrations seem to be relatively insensitive to changes in discharge, a review of the hydrological routine in PLUS+ needs to be undertaken to assess the effect of climate /land use change on compliance with EU Directives.
- The validation dataset needs to be expanded to cover a wider number of years, because SEPA measurements for 2014 only are considered here.

In addition to the above, it is important to continue working with regulatory agencies to ensure that the development and application of PLUS+ is fit for purpose, and that its limitations are understood. We have demonstrated from the case studies that, with good quality site-specific information, PLUS+ performs well. However, until national scale datasets on WWTW discharges, fish farms, etc. are available, the national model should be used with caution.

## Figures and Tables

**Table 1:** Land cover and slope TP export coefficients (kg/ha/yr) from Ferrier et al. [21].

Land Cover Category	Slope Threshold		
	Low 0–4 Degrees	Medium 4–13 Degrees	High 13+ Degrees
Water	0.135		
Wetland	0.085		
Blanket bog & peatland	0.020	0.030	0.040
Arable	0.740	1.140	1.540
Improved grassland	0.320	0.470	0.620
Coarse grassland, Smooth grassland, Heather all types, Bracken	0.060	0.090	0.115
Cliffs			0.045
Montane vegetation	0.025	0.040	0.060
Low scrub	0.140	0.190	0.240
Broadleaved woodland	0.150	0.215	0.275
Mixed woodland, Open canopy young plantation	0.125	0.175	0.235
Coniferous plantation	0.100	0.145	0.195
Recently ploughed land	0.560	0.760	0.960
Woodland recently felled	0.360	0.560	0.760
Ripping	0.300	0.365	0.425
Estuary, Salt marsh, Dune land, Maritime grasslands & heaths	0.000	0.000	0.000
Airfields, Recreational land, Quarries, Other land, Road & rail	0.000	0.000	0.000
Factories & urban	1.380	2.105	2.830

**Table 2:** Sensitivity of water body total phosphorus to export coefficient ranges.

Results for Measured Catchments	P Concentration (µg/l), n = 323		
	Minimum	Median	Maximum
Lowest TP	1.5	2.1	2.6
Mean TP	10.3	12.6	14.8
Highest TP	459.4	473.4	487.3
	Comparison to median export coefficient (model default)		
Lowest TP	71.4%	-	123.8%
Mean TP	81.5%	-	117.4%
Highest TP	97.0%	-	102.9%

**Table 3:** Sensitivity analyses of the effect of changes in land cover and discharge on total phosphorus concentration (µg P/l) for 8030 standing waters.

	Baseline	Land Cover		Discharge (Using 2015 Land Cover)			
		1988	2015	Minus 10%	Minus 5%	Plus 5%	Plus 10%
Minimum	0	0	0	0	0	0	0
5th percentile	3.1	2.4	3.1	3.2	3.1	3	2.9
10th percentile	3.5	2.9	3.5	3.8	3.7	3.4	3.3
median	5.6	4.9	5.6	6	5.8	5.5	5.3
Mean	18.1	17.6	18.1	19.4	18.8	17.6	17
90th percentile	19.7	19.5	19.7	21.1	20.4	19.1	18.5
95th percentile	35.2	36.8	35.2	37.9	36.5	34	32.8
Maximum	3467	3468.9	3467	3722.5	3589.3	3354.1	3249.6
Standard Deviation	104.99	105.33	104.99	112.80	108.72	101.55	98.38
Standard Error	1.172	1.175	1.172	1.259	1.213	1.133	1.098

**Table 4:** Matrix of the number (and percentages) of modelled and measured standing waters within each WFD class.

Measured	High			7 (2.2%)	19 (5.9%)	146 (45.2%)
	Good			13 (4.0%)	21 (6.5%)	53 (16.4%)
	Moderate	1 (0.3%)	2 (0.6%)	6 (1.9%)	14 (4.3%)	22 (6.8%)
	Poor	3 (0.9%)	1 (0.3%)	2 (0.6%)	5 (1.5%)	4 (1.2%)
	Bad	1 (0.3%)	2 (0.6%)		1 (0.3%)	
		Bad	Poor	Moderate	Good	High
				Modelled		

**Table 5:** Total P sources and annual loads (kg) for the Lake of Menteith used in the 1995 model calibration.

Source	Load Min.	Load Max.	Load Median
Land cover	343	665	504
Fish farm	14	30	22
Fish cages	85	105	95
Hotel ^1^	3	13	8
Septic tanks	40	80	60
Birds	25	95	60
Rain	25	75	50
**Sum**	**535**	**1063**	**799**

^1^ [44] states that this source has since been diverted out of the catchment.

## Supplementary Materials

The following are available online at https://www.mdpi.com/1660-4601/17/2/391/s1, S1: Phosphorus Load and Total Phosphorus Calculation in PLUS+; S2: Calculation of surface water total P concentration; S3: PLUS+ User Interface; S4: Per capita sources of P; S5: WFD Breakpoints and Thresholds; S6: Processing Environment, Data Tables and Software; S7: Export coefficients; S8: Land Cover Sensitivity.
