# Supplementary material for: An Assessment of the Performance of the PLUS+ Tool in Supporting the Evaluation of Water Framework Directive Compliance in Scottish Standing Waters
Source: Int J Environ Res Public Health. 2020 Jan 7;17(2):391. doi: 10.3390/ijerph17020391 (PMC7014340; doi:10.3390/ijerph17020391)
Supplement: Supplementary file 1 [file ijerph-17-00391-s001.pdf]

Supplementary Information

1 Phosphorus Load and Total Phosphorus Calculation in PLUS+

The structure of the TP and P calculation in PLUS+ is shown in Figure 1.

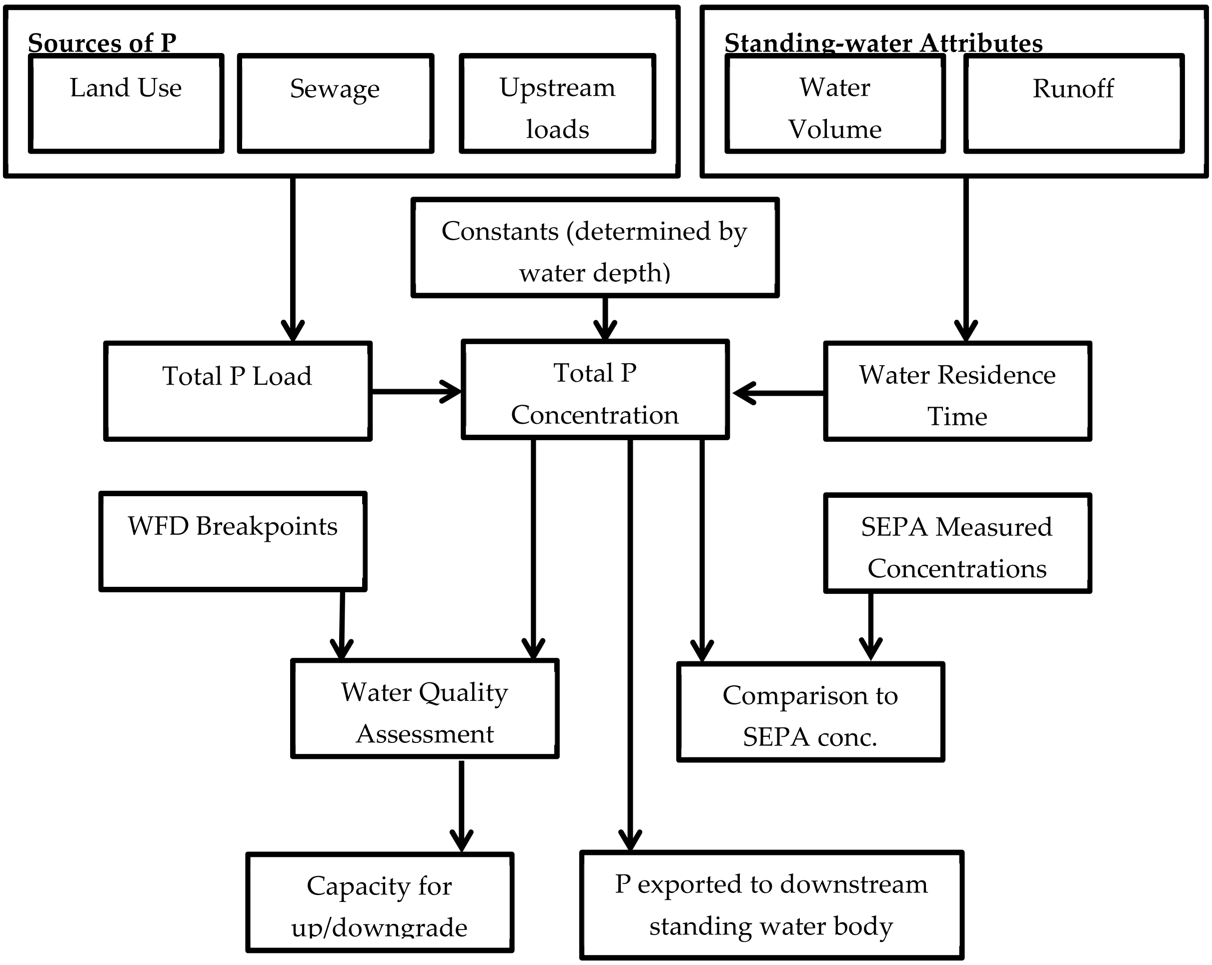

Figure 1. PLUS+ TP and P sources.

The default model output is based on the 2011 census data for which a population density of 0.3 people per hectare has been used to define a threshold between urban and rural population. All waste water for urban populations is assumed to reach the surface water body via waste water treatment works (WWTW) and a per capita P load rate (loss coefficient) has been defined by default as 0.9125 kg P per capita. Waste water from rural populations is assumed to enter the surface water body via septic tanks and a per capita P load rate (loss coefficient) has been defined as a default value of 0.25 kg P per capita.

Table 1 Default point source values

Rural/urban population threshold	0.3 per hectare
Urban Loss Coefficient	0.9125 per capita
Rural Loss Coefficient	0.25 per capita

It is assumed that the entire population within the local catchment and only the population within the local catchment contributed P to the surface water. Population in the network of connected

catchments is routed via any upstream linked surface water body using the FlowRouting table LoadPrecursor and information in the LocalCatchment_and_Network feature class to identify the flow network. Please refer to Section 6 for descriptions of the tables used in PLUS+. The actual location of the WWTW is not modelled in PLUS+.

2 Calculation of surface water total P concentration

The overall total P load to a waterbody is calculated from summing the estimates of point sources, land cover total P load (see section 2.3.1 and 2.3.2) and P from upstream standing water bodies. This total load is used to calculate a total P concentration using OECD (Eutrophication of Waters, *Paris 1982*) equations (equation 1).

$$[TP] = a\left(\frac{J/R}{1+\sqrt{T_W}}\right)^b$$

(Equation 1)

where [TP] is the standing water body total P concentration in micrograms per litre (μg/l), J is the sum of inputs of P in milligrams per year (mg/yr), R is the runoff (Equation 3), T_W is the water residence time in years and a and b are model parameters.

For standing water bodies with a mean depth of >= 3 m, (a, b) = (1.55, 0.82). For shallow standing water bodies with a mean depth < 3 m, (a, b) = (1.02, 0.88). The values a and b for each water body are read from the LoadPrecursor table (see Section 6). Measured or modelled mean depths are available for all standing water bodies are also in the LoadPrecursor data set.

The water residence time, T_W is calculated as follows:

$$T_W = \frac{A\bar{z}}{R}$$

(Equation 2)

where A is the area of the standing water body in square metres (m^2), $\bar{z}$ is the mean depth in meters and R is the runoff in cubic metres per year (m^3/yr). Runoff is available for all standing water bodies in the database. It is calculated in the following manner:

$$R = jA_L + \sum R_{Up}$$

(Equation 3)

where j is the local runoff in m/yr (this value was taken from the model NIRAMS [Dunn 2004]), A is the local catchment area in m^2 and sum of R_{Up} is the sum of upstream runoff which can be calculated from upstream inflows.

The sum of inputs of phosphorus, J, is calculated in the following manner:

$$J = A_L \sum p_i L_i + s + x + y + \sum R_{Up}[TP]_{Up}$$

(Equation 4)

where the first term is the sum of the different phosphorus loads for the different proportions of land cover classes, s is the sewage load (WWTW and septic tanks), x is the sum of any additional point source inputs and y is an alteration that a user may wish to make when running a scenario. The last term is the sum of upstream inputs. The equation is solved in a cascading manner, starting with 0

Strahler stream order standing waters and proceeding down a chain of connected catchments (each order summing the relevant and topologically determined upstream orders), making it possible to simulate TP concentrations for a series of connected standing water bodies.

The OECD equations used in PLUS+ are only valid for standing waters where annual TP load and annual turnover control the observed TP (OECD, 1982).

3 PLUS+ User Interface

The tool is designed to be used in a production environment using Esri ArcGIS. The tool has flexible inputs of data sets through pull-down menus, management of scenarios through tabbed windows and it provides the user with full control of the model parameters. This is described in detail in the user guide [1].

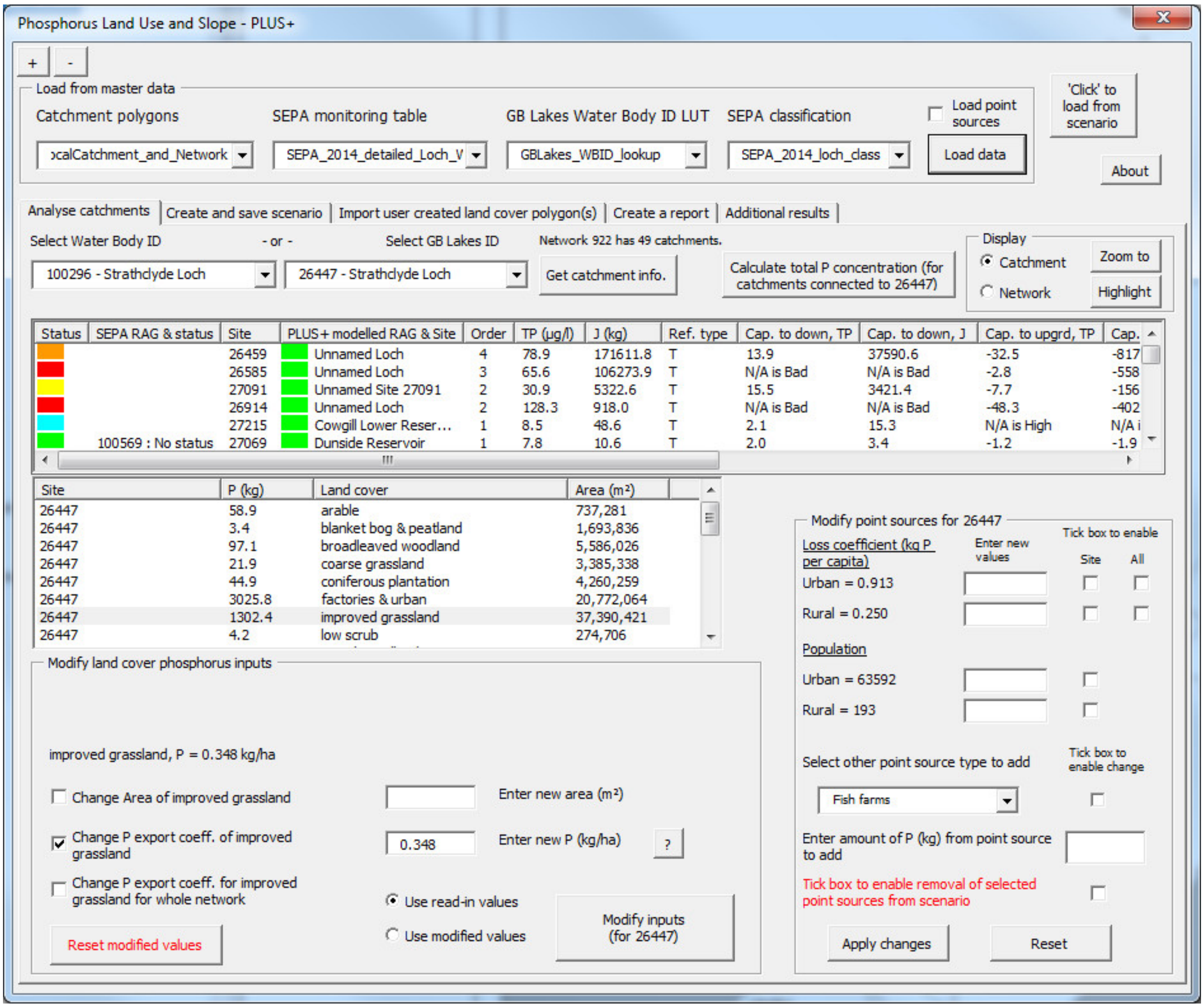

Figure 2. The main PLUS+ user interface

PLUS+ uses WFD water quality breakpoints and the SEPA red-amber-green traffic light system to alert the user to how close water bodies' TP concentration is to a water quality boundary (e.g. from moderate to poor) and their capacity for the uptake of additional P before status is downgraded to a lower category. PLUS+ also calculates how much P would be required to be removed from a water body for the status to be improved. In doing these calculations PLUS+ takes account of in-flows and out-flows to the catchment being analysed.

4 Per capita sources of P

The UK 2011 census data was used as the primary source of information for determining the location and amount of P from waste water treatment plants and sceptic tanks.

Data zone boundaries (the key geography for the dissemination of small area statistics in Scotland) and Ordnance Survey MasterMap residential address locations (the most comprehensive address location data) were used to calculate a mean count of persons per residential property for each data zone. A raster population density map was then created from the residential property locations and the calculated mean number of persons per property from the appropriate data zone. PLUS+ differentiates between urban and rural per capita P exports and uses a threshold of 0.3 persons per hectare as a population density boundary. Following trials with a range of mapping unit sizes, a grid of 500 m (25 Ha) was chosen to aggregate the population to determine whether the locations were urban or rural. While this was found to result in many small hamlets being described as urban it was the minimum resolution required to provide an acceptable degree of precision for the depiction of larger urban units.

Table 2 Comparison of populations in standing water catchments included in the PLUS+ model using data from the 2001 and 2011 censuses.

Class	2001	2011 with raster density surface based on populations in 500m cells
Urban	757,174	865,131
Rural	38,898	30,730
Total	796,072	895,861

Note that according to official census data Scotland's total population rose from 5,062,000 in 2001 to 5,295,000 in 2011.

This revision to the population data has resulted in an increase to the modelled P load of the 8030 sub-catchments of 96,000 Kg compared to the previous version of PLUS+. The largest increase is in the sub-catchment from south-west of Edinburgh to Leith docks which has seen its urban population increase from 181,585 to 198,819 with a resulting increase in P load of 15,702 kg. Of the 8030 sub-catchments 1,043 (13 %, mean increase 99.3 kg) have seen their P load increase and 993 (12 %, mean reduction 7.2 kg) have seen their P load decrease as a result of the updating of the population model.

5 WFD Breakpoints and Thresholds

WFD breakpoints are used to determine the water body status – High, Good, Moderate, Poor, Bad.

The WFD breakpoint data is supplied by SEPA.

Table 3 WFD Breakpoints – a sample of the 324 different groups of values

High/Good	Good/Moderate	Moderate/Poor	Poor/Bad
5.000	8.000	16.000	32.000
5.017	8.000	16.000	32.000
5.079	8.000	16.000	32.000
5.118	8.000	16.000	32.000

5.123	8.061	16.122	32.243
5.223	8.161	16.323	32.646
5.232	8.314	16.629	33.257
5.243	8.000	16.000	32.000
5.263	8.087	16.174	32.348
…			
46.127	61.070	122.139	244.279
46.201	64.236	128.472	256.944

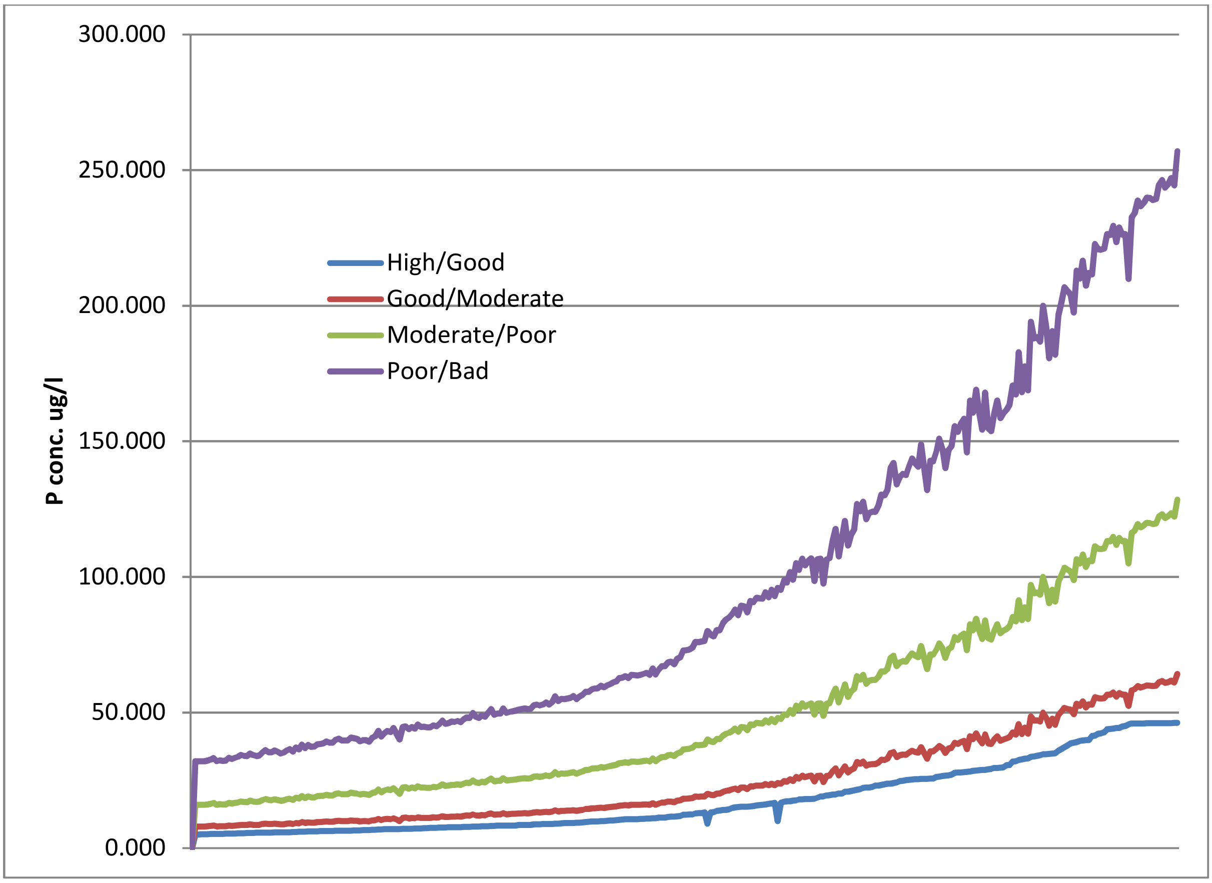

Figure 3 Distribution of TP break point values

The Scottish Environment protection Agency (SEPA) uses a traffic light system of red/amber/green to highlight catchments with a P concentration which is close to a threshold for a less favourable condition (status). The PLUS+ display of the status of each sub-catchment includes a graphic showing the P concentration of the selected catchment with its current calculated status and showing the distance to the higher and lower thresholds (Figure 4). If a concentration is within 3 % of the interval between two adjacent class boundaries above the lower bound then it is classed as red. If the concentration is between 3 % and 20 % of the interval above the lower bound then it is classed as orange, otherwise it is classed as green.

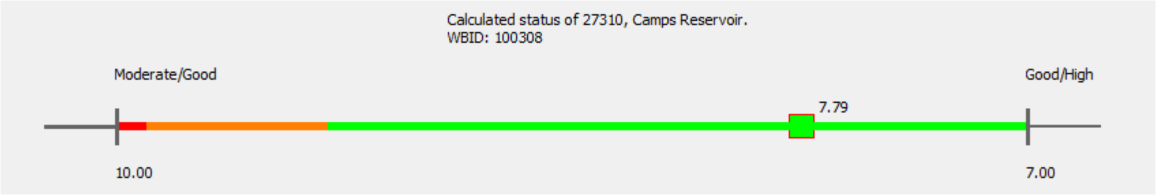

Figure 4 Red/amber/green traffic light system, graphical display from PLUS+

6 Processing Environment, Data Tables and Software

PLUS+ is not a standalone application. It runs within the Visual Basic for Applications environment of Esri ArcMap and leverages ArcMap functionality for data storage and access, spatial processing, data display, and report and map exporting. The PLUS+ software and data tables are available from the GitHub repository https://github.com/DavidHutton/plus [2]. A valid licence for ArcGIS is required to run PLUS+.

PLUS+ has been tested and is compatible with ArcMap versions 9.3 to 10.5. The data is stored in proprietary Esri personal geodatabases (PGDB) or file geodatabases (GDB). PLUS+ uses two PGDB: one for the source data for running the model and one for scenario data to store user generated scenarios.

SourceData.mdb contains the following tables:

1. CatchmentSewage (Urban and rural populations and their associated P load)
2. CatchP (Land cover type and area and associated P)
3. Exports (P look up table for land cover/slope combinations)
4. FlowRouting (Standing water body flow relationships)
5. LoadPrecursor (Standing water body attributes)
6. PerCapitaTPLoads (P look up table for urban and rural P load)
7. TPBreakPoints (Water quality P break points)

and the following polygon feature classes:

1. LocalCatchment_and_Network (Catchment polygons, and their attributes)
2. SlopeClass_LandCover (Land cover/slope polygons)

Additionally the following tables containing SEPA monitoring information are required:

1. SEPA_detailed_Loch_WB_classification (standing water body classifications for WBID)
2. SEPA_20*XX*_loch_class (measured P concentration for specified years *XX)*

ScenarioData.mdb largely duplicates the SourceData above but with names suffixed with "_S" to denote scenario tables. SlopeClass_LandCover and FlowRouting are not included as they will not be modified. Additionally ScenarioData.mdb includes a table "Scenario". This table contains all the information to identify and describe the scenario:

1. ScenarioID

2. ScenarioName
3. ScenarioCreator
4. ScenarioCreationDate
5. Comment
6. Region

Note that the two SEPA monitoring tables are not duplicated within the scenario structure so that whether the baseline data or scenario data are being loaded the SEPA tables must be selected first. Additionally, the measured values are not incorporated in the PLUS+ modelling calculations, they are included in the reports and output windows for information and comparison with the modelled values. Point sources of P added to the model by a user through PLUS+ will be stored in the scenario table PointSource_S. Any number of point sources may be added by the user (only subject to operating system and database restrictions). This table can also be edited manually to add point sources to a scenario. The scenario database does not include a "FlowRouting" table as the topological relationships of standing water bodies will not be modified by any of the scenario creations.

The Visual Basic source code and the data tables are available in a GitHub repository at https://github.com/DavidHutton/plus [2]. The computer code has comments to guide comprehension and it includes detailed descriptions of the data tables required. These are also described in the user guide [1]. Please note that this software will only run under the Visual Basic for Applications environment of the Esri ArcMap software and that a valid Esri licence is required.

7 Export coefficients

The minimum, maximum and median land cover export coefficients are detailed in Table 4. In sensitivity testing each of the minimum, median and maximum settings were used in model runs (i.e. a run was made using the minimum settings and then using the median settings etc.), in all other analyses the median values were used.

Table 4 Land cover export coefficients (kg/ha/yr)

Land Cover Description	Export coefficient for each slope classification								
	Low slope			Medium slope			High slope		
	Min.	Max.	Med.	Min.	Max.	Med.	Min.	Max.	Media.
water	0.07	0.2	0.135	0	0	0	0	0	0
wetland	0.02	0.15	0.085	0	0	0	0	0	0
blanket bog & peatland	0.01	0.03	0.02	0.02	0.04	0.03	0.03	0.05	0.04
arable	0.4	1.08	0.74	0.74	1.54	1.14	1.08	2	1.54
improved grassland	0.2	0.44	0.32	0.32	0.62	0.47	0.44	0.8	0.62
coarse grassland	0.04	0.08	0.06	0.06	0.12	0.09	0.08	0.15	0.115
smooth grassland	0.04	0.08	0.06	0.06	0.12	0.09	0.08	0.15	0.115
heather all types	0.04	0.08	0.06	0.06	0.12	0.09	0.08	0.15	0.115
bracken	0.04	0.08	0.06	0.06	0.12	0.09	0.08	0.15	0.115
cliffs	0	0	0	0	0	0	0.01	0.08	0.045
montane vegetation	0.01	0.04	0.025	0.02	0.06	0.04	0.04	0.08	0.06

low scrub	0.1	0.18	0.14	0.14	0.24	0.19	0.18	0.3	0.24
broadleaved woodland	0.1	0.2	0.15	0.15	0.28	0.215	0.2	0.35	0.275
mixed woodland	0.08	0.17	0.125	0.12	0.23	0.175	0.17	0.3	0.235
coniferous plantation	0.06	0.14	0.1	0.1	0.19	0.145	0.14	0.25	0.195
recently ploughed land	0.4	0.72	0.56	0.56	0.96	0.76	0.72	1.2	0.96
woodland recently felled	0.2	0.52	0.36	0.36	0.76	0.56	0.52	1	0.76
open canopy young plantation	0.08	0.17	0.125	0.12	0.23	0.175	0.17	0.3	0.235
ripping	0.25	0.35	0.3	0.3	0.43	0.365	0.35	0.5	0.425
estuary	0	0	0	0	0	0	0	0	0
salt marsh	0	0	0	0	0	0	0	0	0
duneland	0	0	0	0	0	0	0	0	0
maritime grasslands & heaths	0	0	0	0	0	0	0	0	0
airfields	0	0	0	0	0	0	0	0	0
recreational land	0	0	0	0	0	0	0	0	0
quarries	0	0	0	0	0	0	0	0	0
factories & urban	0.8	1.96	1.38	1.38	2.83	2.105	1.96	3.7	2.83
other land	0	0	0	0	0	0	0	0	0
road & rail	0	0	0	0	0	0	0	0	0
NULL	0	0	0	0	0	0	0	0	0

8 Land Cover Sensitivity

The land cover model used in this version of PLUS+ was updated in 2015 to use a hybrid data set assembled from the most accurate and recent data then available. This was created from Ordnance Survey MasterMap, the National Forest Inventory (Forestry Commission), the Integrated Agricultural Census Scotland (IACS, Scottish Government), GreenSpace Scotland and Land Cover of Scotland (LCS88, for semi-natural, non-forested environments). The resulting land cover model was compared with the previous version of the land cover model which had been sourced entirely from LCS88. The results of this comparison, and the effects on the export coefficients to be used in PLUS+ is summarised in Table 5. This table shows that 67 % of the land described as "heather moor" in the original land cover (first column) was updated to "coarse grassland" in the revised land cover (second column). While the old and revised land covers did not match (the value in column "Match" = "No") there was no change in the associated P export as the Original to Revised export coefficients are both 0.06 kg/yr/ha (final column).

The impact of change in land cover is discussed in the sensitivity analysis section of the report.

Table 5 Comparison of Original (LCS88) land cover and Revised (2015 Hybrid)

Original (LCS88)	Revised (2015 Hybrid)	Area (Ha)	% of orig. area	Match	Export coefficients: Original to Revised (kg/yr/ha)
heather moor	coarse grassland	1367853	67%	No	0.06 to 0.06
improved grassland	improved grassland	899507	79%		0.32 to 0.32
blanket bog & peatland	coarse grassland	685937	67%	No	0.02 to 0.06
arable	arable	533678	60%		0.74 to 0.74
heather moor	heather moor	508805	25%		0.06 to 0.06
coarse grassland	coarse grassland	309326	83%		0.06 to 0.06
coniferous plantation	coniferous plantation	301445	48%		0.1 to 0.1
blanket bog & peatland	blanket bog & peatland	271677	26%		0.02 to 0.02
arable	improved grassland	269916	30%	No	0.74 to 0.32
montane vegetation	coarse grassland	218116	78%	No	0.025 to 0.06
smooth grassland	coarse grassland	211618	48%	No	0.06 to 0.06
coniferous plantation	mixed woodland	195384	31%	No	0.1 to 0.125
factories & urban	factories & urban	142723	93%		1.38 to 1.38
water	water	142547	93%		0.135 to 0.135
smooth grassland	improved grassland	98048	22%	No	0.06 to 0.32
heather moor	mixed woodland	93755	5%	No	0.06 to 0.125
open canopy young plantation	coniferous plantation	91309	56%	No	0.125 to 0.1
coniferous plantation	ripping	82743	13%	No	0.1 to 0.3
improved grassland	arable	77741	7%	No	0.32 to 0.74
recently ploughed land	coniferous plantation	76468	51%	No	0.56 to 0.1
improved grassland	coarse grassland	71090	6%	No	0.32 to 0.06
smooth grassland	smooth grassland	67542	15%		0.06 to 0.06
montane vegetation	montane vegetation	57139	20%		0.025 to 0.025
open canopy young plantation	mixed woodland	45813	28%	No	0.125 to 0.125
mixed woodland	mixed woodland	45465	48%		0.125 to 0.125
recently ploughed land	mixed woodland	43388	29%	No	0.56 to 0.125
broadleaved woodland	broadleaved woodland	37551	35%		0.15 to 0.15
smooth grassland	mixed woodland	35615	8%	No	0.06 to 0.125

improved grassland	mixed woodland	34354	3%	No	0.32 to 0.125
heather moor	improved grassland	33305	2%	No	0.06 to 0.32
broadleaved woodland	mixed woodland	29994	28%	No	0.15 to 0.125
coarse grassland	improved grassland	27857	7%	No	0.06 to 0.32
arable	road & rail	26304	3%	No	0.74 to 0
blanket bog & peatland	mixed woodland	24988	2%	No	0.02 to 0.125
broadleaved woodland	coarse grassland	24668	23%	No	0.15 to 0.06
coarse grassland	mixed woodland	23440	6%	No	0.06 to 0.125
improved grassland	factories & urban	21240	2%	No	0.32 to 1.38
cliffs	coarse grassland	20811	53%	No	0 to 0.06
blanket bog & peatland	improved grassland	20761	2%	No	0.02 to 0.32
mixed woodland	broadleaved woodland	19874	21%	No	0.125 to 0.15
recently ploughed land	recently ploughed land	19594	13%		0.56 to 0.56
arable	mixed woodland	19497	2%	No	0.74 to 0.125
arable	factories & urban	19454	2%	No	0.74 to 1.38
other land	coarse grassland	19307	72%	No	0 to 0.06
bracken	coarse grassland	19220	60%	No	0.06 to 0.06
cliffs	cliffs	17513	45%		0 to 0
woodland recently felled	coniferous plantation	17366	56%	No	0.36 to 0.1
recreational land	recreational land	16726	88%		0 to 0
heather moor	coniferous plantation	16219	1%	No	0.06 to 0.1
heather moor	smooth grassland	14467	1%	No	0.06 to 0.06
coniferous plantation	recently ploughed land	13768	2%	No	0.1 to 0.56
open canopy young plantation	open canopy young plantation	13648	8%		0.125 to 0.125
mixed woodland	coniferous plantation	12121	13%	No	0.125 to 0.1
improved grassland	road & rail	10890	1%	No	0.32 to 0
duneland	duneland	10098	42%		0 to 0
coniferous plantation	coarse grassland	10035	2%	No	0.1 to 0.06

9 References

1 Donnelly, D.; Booth, P.; Ferrier, R.C.; Stutter, M. (2011). Phosphorus Land Use and Slope (PLUS+) Model

User Guide & Computer Code. *Internal report produced on behalf of the Scottish Environment Protection Agency and The James Hutton Institute* ***2011***
(http://www.hutton.ac.uk/sites/default/files/files/Plus+%20User%20Guide%20-%20v1_6.pdf)
2 Donnelly, D. 2019. Phosphorus Land Use and Slope (PLUS+) Model (Data) & Computer Code. *Git Repository*. (https://github.com/DavidHutton/plus)
